# Effect of varying anchorage force intensity on upper molar distalization using clear aligners: a finite element study

**DOI:** 10.1590/2177-6709.30.6.e2525313.oar

**Published:** 2026-02-09

**Authors:** Douglas Teixeira da SILVA, Weber José da Silva URSI, Carlos FLORES-MIR, Ki Beom KIM, Guilherme de Araújo ALMEIDA

**Affiliations:** 1 Federal University of Uberlândia, School of Dentistry, Department of Pediatric Dentistry and Orthodontics (Uberlândia/MG, Brazil).; 2 São Paulo State University, School of Dentistry, Department of Dentistry and Dental Materials (São José dos Campos/SP, Brazil).; 3 University of Alberta, School of Dentistry, Department of Dentistry and Dental Hygiene (Edmonton, Canada).; 4 Saint Louis University, Center for Advanced Dental Education Saint Louis, Department of Orthodontics (Missouri, USA).

**Keywords:** Finite element method, Clear aligner, Malocclusion, Angle class II, Análise de elementos finitos, Alinhadores transparentes, Má oclusão Classe II de Angle

## Abstract

**Introduction:**

This study investigated the effects of maxillary molar distalization using clear aligners and skeletal anchorage, specifically examining the influence of varying anchorage force levels through 3D finite element modeling.

**Method:**

Eight models were developed, systematically varying anchorage force (1.66 N and 3.34 N) from infrazygomatic crest (IZC) screws, force application site (precision cuts or buttons), and the presence or absence of vertical rectangular attachments. A 0.2 mm activation was applied between the first and second molars.

**Results:**

Results indicated that variations in anchorage force did not significantly alter the displacement of the second molars or anterior teeth (canines and central incisors) across the X, Y, and Z axes, provided the force application site and attachments remained constant. However, changing these variables led to observable differences in displacement. Notably, models employing precision cuts and vertical attachments showed second molar distalization, expansion, and extrusion. Additionally, canines displayed reduced mesial crown displacement and intrusion, while central incisors moved labially with less intrusion.

**Conclusions:**

Overall, none of the combinations tested were sufficient to prevent some anchorage loss or unwanted tooth movement. Variations in anchorage force did not significantly affect the extent of second molar distalization or anterior anchorage loss. However, the precision cuts and vertical attachments on molars and premolars resulted in different and more pronounced unwanted displacements.

## RESUMO

**Introdução:**

Este estudo investigou os efeitos da distalização de molares superiores utilizando alinhadores transparentes e ancoragem esquelética, examinando especificamente a influência da variação dos níveis de força de ancoragem, por meio de elementos finitos.

**Método:**

Oito modelos foram desenvolvidos, variando sistematicamente a força de ancoragem (1,66 N e 3,34 N) dos parafusos na crista infrazigomática (IZC), o local de aplicação da força (*precision cuts* ou botões) e a presença ou ausência de *attachments* retangulares verticais. Uma ativação de 0,2 mm foi aplicada entre o primeiro e o segundo molares.

**Resultados:**

Os resultados indicaram que variações na força de ancoragem não alteraram significativamente o deslocamento dos segundos molares ou dos dentes anteriores (caninos e incisivos centrais) nos eixos X, Y e Z, desde que o local de aplicação da força e os *attachments* permanecessem constantes. No entanto, a alteração dessas variáveis levou a diferenças observáveis no deslocamento. Notavelmente, os modelos que empregaram *precision cuts* e *attachments* verticais apresentaram distalização, expansão e extrusão do segundo molar. Além disso, os caninos apresentaram redução no deslocamento para mesial da coroa e na intrusão, enquanto os incisivos centrais moveram-se vestibularmente com menor intrusão.

**Conclusões:**

De modo geral, nenhuma das combinações testadas foi suficiente para evitar alguma perda de ancoragem ou movimentação dentária indesejada. Variações na força de ancoragem não afetaram significativamente a extensão da distalização do segundo molar ou a perda de ancoragem anterior. No entanto, os *precision cuts* e os *attachments* verticais em molares e pré-molares resultaram em deslocamentos indesejáveis distintos e mais pronunciados.

## INTRODUCTION

Technological and material advancements have expanded clear aligner capabilities to treat complex malocclusions, including those requiring extractions, molar distalization, and orthognathic surgery. Non-extraction Class II malocclusions, which require molar distal movement, remain challenging for both fixed appliances and aligners. In non-growing patients, aligner therapy typically involves staged movements, varying with orthodontist preference.^1^ ^,^ ^2^ Commonly, first molars begin distalization once second molars reach about half to two-thirds of their target position, with subsequent teeth following in a similar sequence. However, aligner-generated distal forces often result in anchorage loss, leading to increased overjet. Studies show that bodily molar distal movement with aligners is limited to 2-3 mm and often accompanied by tipping.^3^

Class II elastics are widely used to mitigate anchorage loss.^3^ These are attached via precision cuts directly on aligner or bonded buttons, requering patient compliance-up to 22 hours of daily wear for several months. Temporary anchorage devices (TADs) offer superior anchorage control compared to Class II elastics, with both fixed appliances^4^ and aligners.^5^ ^,^ ^6^ The infrazygomatic crest (IZC) is a common TAD site, allowing near-vertical insertion that reduces root contact risk.^7^ However, questions remain: Does increasing anchorage force from IZC screws to buttons or precision cuts improve anchorage without distorting the aligner? How do vertical attachments affect the force system and resulting tooth movements?

Literature suggests various force levels for molar distalization: 100g,^8^ 150g,^9^ 200g,^8^ ^,^ ^10^ or ranges like 1-3N,^11^ 100-200g,^1^ and 1.5-3.0N.^12^ Cui et al.^10^ suggested that higher force may improve bodily movement. Vertical rectangular attachments are recommended to reduce molar distal tipping.^13^ Anchorage location-whether using precision cuts or buttons-is also considered critical.^3^ ^,^ ^12^ ^,^ ^14^ ^,^ ^15^

This study used finite element analysis (FEA) to examine the effects of two force intensities: 1.66 N, based on the average force from Class II elastics,^9^ ^,^ ^12^ and 3.34 N, representing an average force for en-masse retraction using an extra-alveolar screw;26(Two levels were tested: 1.66 N, based on the average force from Class II elastics,^9^ ^,^ ^12^ and 3.34 N, representing an average force for en-masse retraction using an extra-alveolar screw.^16^ ^,^ ^17^ applied from TADs to buttons or precision cuts. It also assessed how rectangular vertical attachments, designed to control molar tipping, influence the force system.

## METHODS

This study received ethical approval from the Research Ethics Committee (CEP) of Federal University of Uberlândia (UFU), Brazil (CAAE: 68334822.0.0000.5152), and was registered with the National Research Ethics Council (CONEP, Brazil). A cone-beam computed tomography (CBCT) scan from an 18-year-old Caucasian female with Class II, division 1 malocclusion, healthy periodontium, and a full permanent dentition (excluding third molars) was utilized. Pre-treatment record usage was consented to, with image files stored at the Dental School of the Federal University of Uberlândia, Brasil. 

Cone-beam computed tomography (CBCT) images were acquired using an i-CAT® Classic tomograph with the following parameters: 90 kV, 15 mA, a 10.5 cm × 13.0 cm field of view, 0.18 mm slice thickness, and 0.3 mm voxel dimensions. The effective radiation dose was 125 μSv, and the scan duration was 4.5 seconds.

These images were then imported into Mimics software (version 18.0; Materialise, Leuven, Belgium) for anatomical segmentation. The maxilla was delineated from the incisal edges of the teeth to the zygomatic bone. Maxillary structures, including cortical bone, trabecular bone, enamel, dentin, pulp, and the periodontal ligament, were identified based on image density.^18^ ^,^ ^19^ A 0.2 mm thick layer representing the periodontal ligament^18^ was created around each root using Boolean operations.^18^

Following segmentation, 3D triangular surface models of each maxillary structure were exported in Stereo Lithography (STL) format. The aligner, precision cuts, buttons, and non-beveled rectangular attachments (3 mm height, 1 mm thickness, and 2 mm width) were designed using 3-Matic software (version 18.0; Materialise, Leuven, Belgium). A 12x2 mm extra-alveolar mini-implant (Peclab, Belo Horizonte, Brazil; extra-alveolar mini-implant 5593) STL file was positioned on the zygomatic crest, 11 mm superior to the mesiobuccal cusp of the upper second molar and 70º of angulation with the occlusal plane.^7^

**Figure 1:**
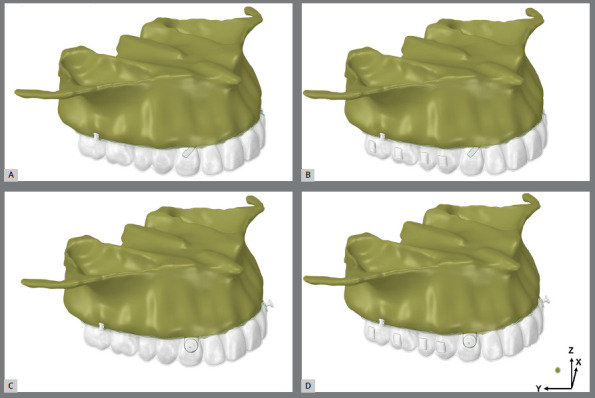
Finite element models with no attachments and with precision cuts (**group A**) models 1, 2) or buttons (**group B**) models 3, 4), and with vertical rectangular attachments and precision cuts (**group C**) models 5, 6) or buttons (**group D**) models 7, 8). Anchorage forces were 1.66 (models 1, 3, 5, 7) and 3.34 N (models 2, 4, 6, 8). Representation of X, Y, and Z axes.

The aligner material was 0.75 mm thick. Precision cuts or bonded buttons were placed at the mid-cervical region of the upper canines. These STL surface files were imported and merged into MSC Patran® 2010 (MSC Software, Santa Ana, CA, USA), where the model was manually meshed with 10-node tetrahedral elements, creating a volumetric element mesh. This mesh was then imported into FEA software (MSC. Marc/Mentat, MSC Software) for structural analysis. The contact conditions were established using three types of relationships. Bonded relationships were assigned to the bone-to-bone, root-to-periodontal ligament (PDL), PDL-to-bone, and tooth-to-attachment interfaces. A frictional contact (0.2mm.) was used between the teeth and clear aligners, as well as between the attachments and the aligners. All tooth-to-tooth contacts were set to be frictionless (Fig. 1). Nodes on the maxilla-excluding the palate-were fully constrained in the x-, y-, and z-directions. All materials were modeled as linear-elastic, isotropic, and homogeneous, with modulus of elasticity and Poisson’s ratio values defined according to Table 1.^1^ ^,^ ^18^ ^,^ ^20^ ^-^ ^24^

**Table 1:** Isotropic mechanical properties of the models.

Material	Modulus of Elasticity (MPa)	Poisson Coeficient (v)
Aligner	528	0.36
Attachments	12500	0.36
Button	206000	0.3
Cortical Bone	13.700	0.3
Dentin	17070	0.3
Enamel	73720	0.23
Extra-alveolar Screw	103400	0.35
Periodontal Ligament	0.50	0.45
Pulp	2.07	0.45
Trabecular Bone	1.370	0.3

MPa - Megapascal.

Model convergence was verified through mesh refinement, particularly at contact interfaces, geometric discontinuities, and in the upper molars, canines, and incisors. The refinement process was continued until the results became independent of the number of elements.

A FEA simulation was conducted using static loading.^1^ The step distance for molar distalization was set at a 0.2 mm displacement along the positive y-axis of original model. Subsequently, a clear aligner under loading conditions. The loading force was then applied due to the mismatch between the aligner and the original model. 

Eight models were then generated, systematically varying the force application site (buttons or precision cuts), anchorage force magnitude, and the presence of vertical rectangular attachments (Fig. 1):

Group I

Model 1: No attachments, 1.66 N force to precision cuts.

Model 2: No attachments, 3.34 N force to precision cuts.

Model 3: No attachments, 1.66 N force to buttons.

Model 4: No attachments, 3.34 N force to buttons.

Group II

Model 5: Non-beveled vertical rectangular attachments at the center of molar and premolar crowns, 1.66 N force to precision cuts.

Model 6: Non-beveled vertical rectangular attachments at the center of molar and premolar crowns, 3.34 N force from to precision cuts.

Model 7: Non-beveled vertical rectangular attachments at the center of molar and premolar crowns, 1.66 N force to buttons.

Model 8: Non-beveled vertical rectangular attachments at the center of molar and premolar crowns, 3.34 N force to buttons.

To validate the model, a three-step coherence analysis was performed to ensure the expected direction and distribution of stresses. First, this analysis confirmed the expected direction and stress distribution. The second step involved superimposing an initial maxilla scan on two subsequent 14-day interval scans, which incorporated a 0.2 mm interproximal separator between the upper molars and used the palatal rugae as a reference.^25^ The displacement of the second molar, canine, and upper central incisor was then measured and compared with the results from the finite element model. Finally, the root mean squared error (RMSE) was calculated between the displacement of the upper second molar obtained in the scan and in the finite element models.

Each model comprised an average of 753,406 nodes and 472,591 elements-data analysis involved comparing quantitative scales generated within the models and results from specific areas of interest. The modified Von Mises criterion was used to process results, displaying maximum and minimum stresses as tensile and compressive forces, respectively, measured in megapascals (MPa). 

Displacements were measured at the mesiobuccal cusp tip of the second molar, the canine tip, and the incisal edge of the upper central incisor. These measurements assessed the initial orthodontic movement tendencies of these teeth during second molar distalization (Table 2). Displacements were represented by vector arrows, indicating movement direction and intensity in the X, Y, and Z axes planes, and quantified in millimeters (mm) (Fig. 1). The percentage contribution of each tooth’s displacement was determined by its movement in relation to the total absolute displacement of the second molar, canine, and central incisor (Table 3).

**Table 2:** Immediate displacement tendency (mm) of upper second molars, canines, and central incisors of each model (1-8), according to X, Y, and Z axes.

Force (N)	Attachment	Upper Second Molar			Canine			Central Incisor		
		X	Y	Z	X	Y	Z	X	Y	Z
G1 1.66 + PC	Without Attachments	0.34e-2	5.8e-2	0.15e-2	2.1e-2	-1.2e-2	2.1e-2	1e-2	-0.4e-2	0.9e-2
G2 3.34 + PC	Without Attachments	0.34e-2	5.8e-2	0.15e-2	2.1e-2	-1.1e-2	2.1e-2	1.1e-2	-0.4e-2	0.9e-2
G3 1.66 + Button	Without Attachments	0.3e-2	5.7e-2	0.1e-2	2,1e-2	-1.3e-2	2.1e-2	1e-2	-0.4e-2	0.9e-2
G4 3.34 + Button	Without Attachments	0.3e-2	5.8e-2	0.1e-2	2.2e-2	-1.2e-2	2.1e-2	1e-2	-0.3e-2	0.9e-2
G5 1.66 + PC	Vertical	0.8e-2	6.3e-2	-2.1e-2	0.3e-2	-1.4e-2	0.7e-2	-0.8e-2	-0.4e-2	0.5e-2
G6 3.34 + PC	Vertical	0.8e-2	6.3e-2	-2.1e-2	0.3e-2	-1.3e-2	0.7e-2	-0.8e-2	-0.4e-2	0.5e-2
G7 1.66 + Button	Vertical	0.3e-2	5.7e-2	0.1e-2	2.2e-2	-1.4e-2	2.1e-2	1e-2	-0.5e-2	0.9e-2
G8 3.34 + Button	Vertical	0.3e-2	5.7e-2	0.1e-2	2.2e-2	-1.3e-2	2.1e-2	1e-2	-0.5e-2	0.9e-2

**Table 3:** Comparison (%) between the second molar movement and anchorage loss of canine and central incisor.

Model	Upper tooth movement (%)			Anchorage loss (%)
	Second Molar	Canine	Central Incisor	Canine + Central Incisor
1	+78.38%	-16.22%	-5.4%	-21.62%
2	+79.45%	-15.07%	-5,4%	-20.55%
3	+77.03%	-17.56%	-5.41%	-22.97%
4	+79.45%	-16.44	-4.11%	-20.55%
5	+77.78%	-17.28%	-4.94%	-22.22%
6	+78.75%	-16.25%	-5%	-21.25%
7	+75%	-18.42%	-6.58%	-25%
8	+76%	-17.33%	-6.67%	-24%

## RESULTS

The RMSE obtained was 5.62e-3, demonstrating a high degree of adherence to the validation data.

**Figure 2:**
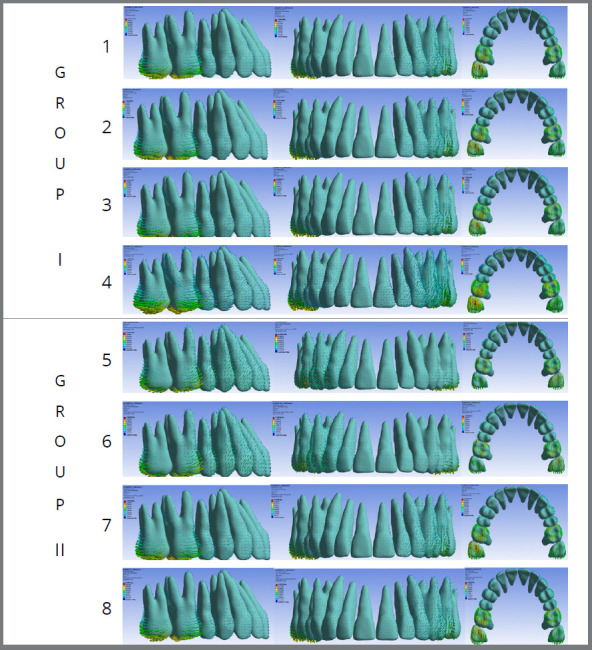
Displacement with aligners: Group I (1, 2, 3, and 4) used no beveled rectangular vertical attachments, while Group II (5, 6, 7, and 8) used them. All groups used anchorage from extra-alveolar screws to either precision cuts (Groups 1, 2, 5, 6) or buttons (Groups 3, 4, 7, 8). Forces of 1.66N (Groups 1, 3, 5, 7) or 3.44N (Groups 2, 4, 6, 8) were applied.

**Figure 3:**
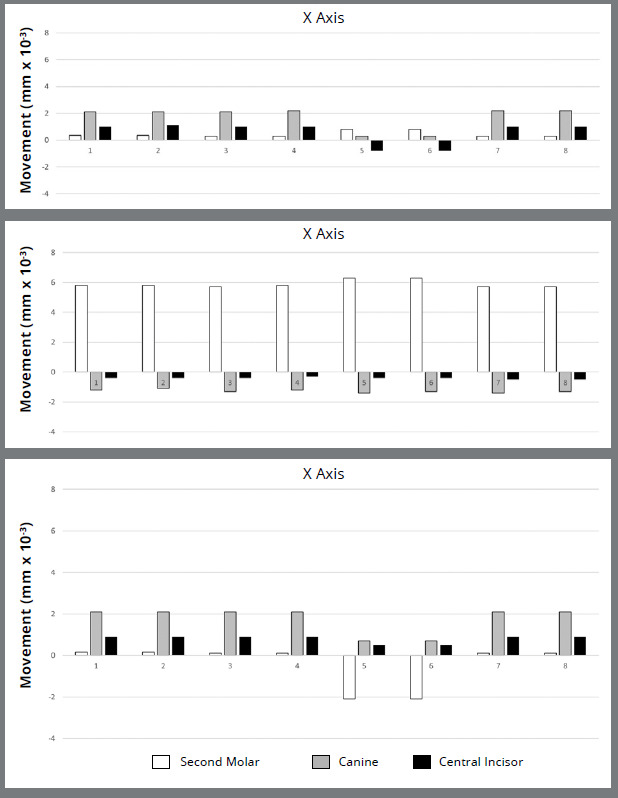
Displacement of second molars, canines, and upper central incisors along the X-axis (positive values represent displacement towards the mid-sagittal plane, negative values away from it), the Y-axis (positive values represent distal displacement, negative values mesial displacement), and the Z-axis (positive values represent intrusive displacement, negative values extrusive displacement).

In the X-axis (Fig. 1, 2, and 3; Table 2) in six models, canines showed the most significant displacement tendency towards the mid-sagittal plane, followed by central incisors and second molars. However, in Models 5 and 6 (non-beveled vertical rectangular attachments and precision cuts), molars and canines displacement towards the mid-sagittal plane decreased. At the same time, incisors showed a tendency to displace away from it. Anchorage force did not significantly influence this displacement.

**Figure 4:**
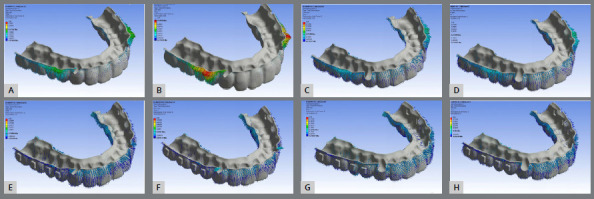
Deformation of aligners without (**A, B, C, D**) and with (**E, F, G, H**) attachments. An anchorage force of 1.66N (**A, C, E, G**) or 3.34N (**B, D, F, H**) was applied from the extra-alveolar screw to either precision cuts (**A, B, E, F**) or buttons (**C, D, G, H**).

Similar results were observed along the Y-axis across all eight models, with comparable distal molar movement and anchorage loss. No combination of force intensity, attachment presence, or force application site (precision cuts or buttons) demonstrated superiority. Mesial movement of the anterior teeth, particularly the canines, was evident in all models (Table 2; Fig. 1, 2, 3 and 4).

Analysis of total displacement distribution revealed that second molars displaced an average of 77.73%, while anchorage loss accounted for 22.27% (16.82% for canines and 5.45% for central incisors (Table 3). This distribution remained consistent regardless of the applied force (1.66 N or 3.34 N), force application site (buttons or precision cuts), or vertical rectangular attachments.

Regarding vertical displacements along the Z-axis (Fig. 4), intrusion was the predominant movement (Table 2, Fig. 2 and 3). Quantitatively, intrusion was minimal in second molars but more pronounced in anterior teeth, especially canines (Models 1, 2, 3, 4, 7, and 8). However, Models 5 and 6 showed reduced canines and central incisors intrusion, accompanied by extrusion of the upper second molars.

Tooth displacement correlated with aligner deformation, which varied depending on the force application site, anchorage force magnitude, and the presence of vertical attachments (Fig. 4 ). In models using precision cuts without attachments (Models 2 and 4), initial deformation primarily occurred in the first and second premolar regions, particularly at the 3.34 N force level (Fig. 5B). When force was applied to buttons without attachments, deformation was distributed throughout the aligner, with a slightly greater concentration in the premolar area (Fig. 4C and D). With vertical attachments, aligner deformation was more uniform, though accentuated near the precision cuts ( Fig. 4E, F, G, and H).

## DISCUSSION

Various strategies have been proposed to minimize or prevent upper molar distalization side effects, including Class II elastics^1^ ^,^ ^14^ ^,^ ^15^ differential tooth movement movements^1^ ^-^ ^3^ ^,^ ^22^ ^,^ ^23^ different attachment designs,^12^ ^,^ ^24^ ^,^ ^26^ and skeletal anchorage.^6^ ^,^ ^10^ ^-^ ^12^ ^,^ ^17^ ^,^ ^27^ Temporary anchorage devices (TADs) offer superior anchorage control over Class II elastics.^5^ ^,^ ^6^ However, optimal force application site (bonded button vs. precision cuts) and force level for maximizing anchorage with minimal side effects remain unclear. Additionally, the influence of attachments on promoting bodily distalization versus undesired tooth movements needs further study-issues addressed in this investigation.

Finite element analysis (FEA), widely used in orthodontics, was applied to simulate and compare the biomechanical effects of molar distalization with clear aligners (CA) under different anchorage forces. Forces were applied from an infrazygomatic crest (IZC) mini-screw to either a precision cut or a bonded button on the canine, with or without attachments on molars and premolars. A 0.2 mm activation between the upper first and second molars was set, following previous studies.^9^ ^,^ ^27^

Intuitively, higher anchorage forces might be expected to produce fewer undesirable side effects on anterior teeth. However, this did not happen; twice the anchorage force failed to yield greater upper second molar distalization or a reduction in anchorage loss for the upper canines and central incisors (Fig. 2 and 3; Tables 2 and 3). Overall, second molars showed distalization, movement toward the midsagittal plane, and intrusion-consistent with prior studies.^9^ ^-^ ^12^ ^,^ ^15^ ^,^ ^24^ Canines and central incisors tended to move mesially, extrude, and shift toward the midsagittal axis, as reported in other studies^2^ (Table 2; Fig. 3).

Molar distal movement and anterior anchorage loss (canines and incisors) were calculated along the Y-axis (mesio-distal), which is critical for Class II correction (Tables 2 and 2). Of the intended 0.2 mm displacement, second molars achieved 77.73% distal movement, with 22.27% anchorage loss (16.82% in canines, 5.45% in incisors). This anchorage loss was less than the 47.14% reported by Liu et al.,^6^ who used a slightly greater distalization force (0.25 mm) on the upper second molars. 

When attachment configuration and force application (buttons or precision cuts) were constant, varying anchorage force had no significant impact on second molars or anterior tooth displacements across all axes (Table 2; Fig. 3). Thus, increasing force did not enhance molar movement. However, altering the force application site and use of attachments led to clear differences.^6^ ^,^ ^10^ ^,^ ^26^ ^,^ ^28^ In Models 5 and 6 (precision cuts with vertical attachments), molars distalized and expanded, but unlike the other groups exhibited extrusion. Canines presented less midline displacement (X-axis) and less intrusion (Z-axis), while incisors moved away from the midline and showed reduced intrusion (Table 2; Fig. 3).

These outcomes may stem from added resistance from attachments combined with force on precision cuts, potentially contributing to side effects. Literature supports attachments for tipping control and aligner retention.^12^ ^,^ ^15^ ^,^ ^24^ ^,^ ^26^ Ayidağa et al.^13^ found vertical rectangular attachments reduce distal tipping, corroborated by Ravera et al.^3^ for displacements up to 2.5 mm.

The question of force application to buttons versus precision cuts has been explored. Our findings align with Ji et al.,^9^ favoring buttons. Conversely, Liu et al.^1^ ^,^ ^6^ and Li et al.^15^ recommended forces to precision cuts, even with Class II elastics. One potential drawback of applying force to a precison-cut on an aligner is that it may negatively impact aligner adaptation, compromising three-dimensional control of tooth movement.

Aligner deformation was assessed based on force application to a precision cut or to a button (Fig. 5). Regardless of the force level, deformation was consistently greater with force applied to precision cuts. It began in the canine cervical area, increased in first premolars, and reduced towards second molars. Applying force to a precision cut may detach the aligner from the tooth surface, compromising the intended tooth movement. ^28^

These results point to three important clinical considerations. First, consistent with the literature^1^ ^-^ ^3^ ^,^ ^8^ ^,^ ^9^ ^,^ ^12^ ^,^ ^14^ ^,^ ^15^, distalization of the upper second molar using aligners does not prevent anchorage loss. This failure appears to occur regardless of increased anchorage force, likely due to an apparent increase in the aligner’s plastic deformation (Fig. 4). Second, it reinforces that in the presence of undesirable effects such as anchorage loss, the use of non-beveled vertical attachments, although intended to improve the control of tooth movements and the retention of the aligners, ends up potentiating them. Third, in these situations of uncontrolled undesirable effects, the insertion of anchorage force in the precision cuts, together with the presence of non-beveled vertical attachments, seems to tend to increase the deformation of the aligner in this region, compromising the efficiency of this type of mechanics.

This study has inherent methodological limitations that may not fully replicate clinical conditions. For instance, this study only evaluated immediate displacement tendencies and did not consider short- or long-term force application; as further displacements occur, the validity of these model assumptions may decrease. Additionally, the FEA model assumes a uniform periodontal ligament (PDL) thickness, which varies in reality. Similarly, because structures such as bones, PDL, and dental structures are often treated as isotropic, clinical extrapolations must be approached with caution.

## CONCLUSION

Overall, none of the combinations tested were sufficient to prevent some anchorage loss or unwanted tooth movement. Variations in anchorage force did not significantly affect the extent of second molar distalization or anterior anchorage loss. However, the precision cuts and vertical attachments on molars and premolars resulted in different and more pronounced unwanted displacements.
